# 

**Published:** 1880-07

**Authors:** 


### CONTENTS FOR JULY, 1880.

ORIGINAL COMMUNICATIONS.

ART. I.—The De Lesseps Canal in its Relation to Hygiene. By G. HALSTED BOYLAND, M. D. 317

ART. II.—Practical Hints Relating to Yellow Fever Prevention. By R. B. S. HARGIS, M. D. .... 334

ART. III.—The Electro-Therapeutics of Displacements of the Uterus. By ED. C. MANN, M. D. .... 343

ART. IV.—Operative vs. Mechanical Dentistry. By Dr. J. ALLEN OSMUN .... 348

Correspondence .... 350

EDITORIAL DEPARTMENT.

Reform in Medical Education .... 352

The Metric System .... 353

Martin's Elastic Bandage .... 353

The American Medical Association .... 355

The Medico-Literary Journal .... 355

Incompatible Medicines .... 356

Medical Rats .... 356

An Original Journal .... 357

Bromide of Ethyl .... 357

Ruth vs. Reuling .... 358

Who are They? .... 358

Criticism .... 359

National Dental Association .... 360

Call for a Mass Convention of the Dentists .... 361

The American Dental Association .... 362

BIBLIOGRAPHICAL DEPARTMENT .... 363

HYMENEAL. NATALITIAL. NECROLOGICAL .... 365

Mortuary Statistics and Meteorological Report .... 366

